# HMGB1, the Next Predictor of Transcatheter Arterial Chemoembolization for Liver Metastasis of Colorectal Cancer?

**DOI:** 10.3389/fonc.2020.572418

**Published:** 2020-12-15

**Authors:** Yuan-dong Sun, Hao Zhang, Ye-qiang Chen, Chun-xue Wu, Jian-bo Zhang, Hui-rong Xu, Jing-zhou Liu, Jian-jun Han

**Affiliations:** ^1^ Interventional Medicine Department, Shandong Cancer Hospital and Institute, Shandong First Medical University and Shandong Academy of Medical Sciences, Ji’nan, China; ^2^ Maternal and Child Health Care Hospital of Shandong Province, Ji’nan, China; ^3^ School of Medicine and Life Sciences, University of Ji’nan-Shandong Academy of Medical Sciences, Ji’nan, China

**Keywords:** liver metastasis of colorectal cancer, drug-eluting beads transarterial chemoembolization, transarterial chemoembolization, high mobility group box 1, HMGB1

## Abstract

HMGB1 is an important mediator of inflammation during ischemia–reperfusion injury on organs. The serum expression of HMGB1 was increased significantly on the 1st day after TACE and decreased significantly which was lower on the 30th day after TACE. Tumor markers of post-DEB-TACE decreased significantly. The correlational analysis showed that patients with low HMGB1 expression had lower risks of fever and liver injury compared those with the higher expression, while the ORR is relatively worse. Patients with lower expression of HMGB1 had longer PFS, better efficacy, and higher quality of life. With the high post-expression, the low expression had lower incidence of fever and liver injury too. There was no statistical difference in the one-year survival among the different groups. The quality of life of all patients was improved significantly. The over-expression of HMGB1 in LMCRC is an adverse prognostic feature and a positive predictor of response to TACE.

## Highlights

The findings of this study show that patients with low expression of HMGB1 before TACE have a lower incidence of severe liver damage. Post-TACE liver damage is proportional to the pre-TACE expression level of HMGB1, and respondents who reported post-TACE lower levels of HMGB1 also reported significantly lower liver damage. The findings from these studies suggest that higher HMGB1 expression levels before TACE may be a prognosis of liver damage and efficacy. Taken together, these results exhibit that patients with severe HMGB1 changes after TACE had more severe liver damage and were less sensitive, but ORR and PFS of them were relatively better. These results confirm that the changes of HMGB1 maybe the predictor of liver damage and efficacy after TACE.

## Introduction

Colorectal cancer is one of the most common malignant tumors in the world, including colorectal cancer and rectal cancer (1). About 10–25% of colorectal cancer patients find simultaneous liver metastasis at the time of diagnosis (2, 3). When patients have distant metastasis outside the primary site, it is difficult to obtain satisfactory results only by surgical resection (4–6). For unresectable metastatic liver cancer, cryoablation, local thermal ablation of liver, transcatheter arterial infusion (TAI), proton therapy, liver radioactive particle implantation, and transcatheter arterial chemoembolization (TACE) are some good non-surgical treatment methods (7–10).

TACE is considered one of the most effective and safe treatment for advanced liver cancer (11). It is generally assumed to play a considerable role in liver solid tumors, but it has also been reported to have the potential to cause significant damage to liver function (12). There are three reasons that liver injury after TACE are common: the history of concomitant cirrhosis, chemotherapeutic drugs, and the process of ischemia–reperfusion in the liver (13). After TACE, the block of blood supply to local liver tissue at the embolic site leads to local ischemia and hypoxia. After a period of time (usually 7–30 days), local blood supply is restored under the dual action of flowing blood and the establishment of collateral circulation. Therefore, we can assume that the liver undergoes a complete ischemia–reperfusion process after TACE. Conventional TACE (c-TACE) and drug-eluting bead transcatheter aterial chemoembolization (DEB-TACE) are widely used at present. Lipiodol suspended with an anticancer drugs and gelatin sponge particles served as embolic-agents are widely used in c-TACE. Chemotherapeutic drugs were delivered to the tumor by super-selective catheterization, and then the nutrient vessels were sealed with embolic materials. At present, several novel spherical embolic drugs-carrying/drug-eluting beads (DEBs) have been developed to release the drug slowly and long-term, reduce liver damage and improve the local concentration of anticancer drugs. The biggest difference between the two is that DEB-TACE combines drugs and embolic materials in drug-loaded microspheres, but their effects on local blood disruption in the liver are similar. Regardless of the difference of treatment modalities, some chemotherapeutic drugs (such as irinotecan, doxorubicin, oxaliplatin, *etc*.) inevitably have a killing effect on peritumoral tissue (14). Lead to the powerful killing effect of chemotherapeutics, normal liver tissues appeared damaged, necrotic, and apoptotic (14). On the other hand, some recent findings show that inflammatory mediators after TACE play a role in the reestablishment of collateral circulation. Therefore, after TACE, timely prediction and clinical treatment of patients’ liver damage can effectively reduce the possibility of tragic outcomes. However, there are certain drawbacks of the current liver function test, like insufficient sensitivity and higher latency. When the results of the liver function test after TACE showed obvious abnormalities, patients often have reached the level of severe liver damage. It is necessary to find a critical demand for prognostic and predictive biomarkers in liver damage (15).

Previous studies in patients with primary liver cancer have shown that expression of high mobility group box-1 (HMGB1) in local liver tissues can rise dramatically in a few hours after TACE (16). Significant changes in serum expression of HMGB1 could be detected at 12 to 24 h after TACE, and it could reach the highest level at 28 to 36 h. Finally, HMGB1 gradually returned to the normal level within the following month (17). In view of the repeated traumatic examination of the liver that will bring certain risks of complication to patients, the concentration level of HMGB1 in the blood is the predictor to analyze the liver damage and avoid the bad impact of repeated liver biopsy. In this study, we studied the level of HMGB1 in the blood after TACE and verified the predictive ability of HMGB1 on liver damage and the efficacy of TACE. To this end, we generated a comprehensive review after TACE at the liver damage, safety and progression-free survival time (PFS) by blood samples, clinical information, and the results of follow-up.

## Method

### Study Design

A prospective, randomized study recruited 106 LMCC patients from December 2017 to July 2019 in Shandong Tumor Hospital as previously described. All procedures were performed with a protocol approved by the ethics committee. Patients were required to be 18 years of age or older and have a diagnosis of liver metastases from colorectal cancer. Patients with a prior anticancer treatment within 2 months were not eligible for enrollment. Prior to the collection of biological samples and TACE, all patients were required to give full informed consent. All patients had radiologic imaging either by computed tomography scanning (CT) and/or magnetic resonance imaging (MRI) before TACE to document the presence of any other metastases. Serum tumor markers (CA19-9,CEA), serum HMGB1 level, liver function and blood cell cluster differentiation antigen were examined 4 to 6 h before TACE and 1, 3, 5, 7, 30 days after treatment. Laboratory analysis of serum preparation was performed at the Shandong Province Cancer Hospital Central Laboratory. Approximately 10 ml of peripheral blood was drawn by the peripheral vein puncture in two standard serum tubes and centrifuged (10 min, 2,000g, room temperature) within 24 h following the collection time to remove clots. The researcher collected and dispensed the serum into multiple 2 ml cryotubes and stored it at −80℃. Any contaminated samples were excluded from the analysis. The concentration of HMGB1 in serum was measured by the ELISA kit (Novus Biologicals, LLC, US) and immune cells were determined by the flow cytometry assay (Thermo Fisher Scientific Inc. US). All assays were run according to the manufacturer’s instructions, and all controls were within the ranges provided by the manufacturer. In this study, all patients underwent TACE for the liver metastases and symptomatic treatment for the possible adverse reaction. Enhanced imaging examination obtained from all cases was centrally reviewed by two radiologists to verify the diagnoses made by the researcher. The patients had a regular review with their physician every month during the first six months and then every two months until the end of follow-up.

The treatments were performed by two designated interventional radiology physicians (20- and 11-years’ experience). The medical imaging results were viewed by a radiologist (minimum 10 years of experience) and reviewed by another radiologist. The follow-up information and results were compiled and maintained by a designated researcher. Data analysis was conducted independently by two researchers.

### Group

Due to the differences in drug release rate and local concentration between the two TACE modalities, we initially divided patients into DEB-TACE (CalliSpheres^®^, Jiangsu Hengrui Medicine Co. Ltd., Jiangsu, P.R. China) group and c-TACE groups. Then, we classified that patients with pre-HMGB1 level in serum above 17.5 pg/ml as the preoperative high expression group and others as the preoperative low expression group. Whether the change of HMGB1 concentration in the sample on the first day after TACE is more than 50% is defined as the grouping standard. According to the high change group of HMGB1 before TACE increased by more than 50%, and the patients with variation less than 50% were low change group.

### Follow-Up

PFS, the most important efficacy indicator in this study, is the time between the date the patient enters the group for treatment and any documented tumor progression or death from any cause (not limited to death from cancer). The treatment outcomes of TACE can be classified as complete response (CR), partial response (PR), stable disease (SD), and progressive disease (PD) according to mRECIST1.1. The objective remission rate (ORR) in this study is the proportion of patients whose target tumor shrinks to the SD level and remains there for a period.

### Statistical Analysis

The statistical data in this study were analyzed using SPSS version 22.0 (SPSS Inc., Chicago, US) and GraphPad Prism 8.0 (GraphPad Software Inc., San Diego, CA 92108). Sample size in this study was calculated by PASS 15.0 (NCSS, LLC. Kaysville, Utah, USA). The sample size was determined by power analysis using preliminary data obtained in our laboratory with the following assumptions: *α* of 0.05 (two-tailed), power of 90%, difference in patients between before and after TACE, and a standard deviation of 17.5 pg/L. Fisher’s exact test (two-tailed) was used to compare categorical variables and Mann–Whitney U test (two-tailed) for continuous variables. PFS was analyzed using the Kaplan–Meier method and compared *via* the log-rank test. Comparisons were made using the log-rank test (for univariate analysis). Between-group comparisons were examined using either the t-test or the chi-square test. The correlation analysis was performed using Poisson’s test, and p-values less than 0.05 were considered statistically significant. All tables are drawn by Microsoft Office Word 2019 (Microsoft, Redmond, WA, USA).

## Results

A total of 126 patients enrolled in the study, and 106 of them (82 males and 24 females) were evaluable. Patients were divided into two groups: 56 of them received DEB-TACE and the rest received c-TACE. The mean age of the evaluable study cohort was 61 years old (range: 30 to 88), with a mean age for the c-TACE group of 60 years old (range: 30 to 79) and DEB-TACE 62 years old (range: 38 to 88). There were 51 patients with rectal cancer (23 c-TACE and 28 DEB-TACE) and 55 patients with colon cancer (27c-TACE and 28 DEB-TACE) diagnosed in the study group. Patient demographics and characteristics are illustrated in **Table 1**. Data analysis was conducted independently by two researchers.

**Table 1:** Patients’ characteristics before TACE.

Characteristics	DEB-TACE	c-TACE
	Patients	Patients
Gender		
Male	41 (73.21%)	41 (82.00%)
Female	15 (26.79%)	9 (18.00%)
Age		
<60	22 (39.29%)	22 (44.00%)
≥60	34 (60.71%)	28 (56.00%)
ECOG Score ^a^		
0	2 (3.57%)	6 (12.00%)
1	28 (50.00%)	27 (54.00%)
2	20 (35.71%)	12 (24.00%)
3	6 (10.72%)	5 (10.00%)
BCLC ^b^		
A	12 (21.43%)	9 (18.00%)
B	44 (78.57%)	41 (82.00%)
Tumor differentiation		
No reported	22 (39.29%)	20 (40.00%)
Low	10 (17.86%)	6 (12.00%)
Moderate	12 (21.43%)	10 (20.00%)
High	12 (21.43%)	14 (28.00%)
The expression of HMGB1 (pg/ml)		
Pre-TACE	19.14 ± 3.91	19.98 ± 3.98
1st after TACE	31.55 ± 7.15	32.86 ± 7.62
3rd after TACE	31.31 ± 7.10	32.55 ± 7.65
5th after TACE	30.75 ± 7.21	32.08 ± 7.52
7th after TACE	28.78 ± 6.69	30.16 ± 6.81
30th after TACE	17.39 ± 2.86	17.01 ± 2.44

^a^ECOG Score：Eastern Cooperative Oncology Group Score Standard.

^b^BCLC: Barcelona Clinic Liver Cancer.

### Level of HMGB1 of Post-TACE

The patients’ average HMGB1 of pre-TACE was 19.68 pg/ml and at 1st after TACE was 32.25 pg/ml, p<0.05. At 30 days after treatment, the level was 17.19 PG/ml, which was statistically significant. The changes of HMGB1 expression in patients are shown in **Table 1**.

### Pre-TACE Level of HMGB1 and Prognosis

The patients were grouped by the level of HMGB1 in the serum before TACE. The basic information about the four groups of patients is shown in **Table 2**.

**Table 2:** Characteristics of patients of HMGB1 expression subgroup.

	DEB-TACE			c-TACE			DEB-TACE			c-TACE		
	High-expression(n = 34)	Low-expression(n = 22)	*P*-value	High-expression(n = 28)	Low-expression(n = 22)	*P*-value	High-change(n = 33)	Low-change(n = 23)	*P*-value	High-change(n = 30)	Low- change(n = 20)	*P*-value
Age	60.47 ± 11.60	64.32 ± 10.15	0.21	58.86 ± 10.59	61.59 ± 12.48	0.41	60.45 ± 10.73	64.17 ± 11.51	0.22	56.90 ± 12.41	64.80 ± 7.87	0.02
Sex(male)	26	15	0.50	22	19	0.49	28	13	0.03	26	15	0.33
Tumor differentiation			0.05			0.66	0.75			0.43		
High	10	2		5	9		7	5		8	6	
moderate	8	4		8	2		7	5		8	2	
low	5	4		5	1		5	5		3	3	
HMGB1 expression(pg/ml)	22.36 ± 2.83	15.79 ± 1.02	<0.01	21.78 ± 2.69	15.19 ± 1.09	<0.01	88.84%	33.64%	<0. 01	87.93%	31.77%	<0.01
BCLC ^a^			0.40			0.45			0.19			0.77
A	6	6		4	5		9	3		5	4	
B	28	16		24	17		24	20		25	16	

^a^BCLC, Barcelona Clinic Liver Cancer.

**Figure 1:**
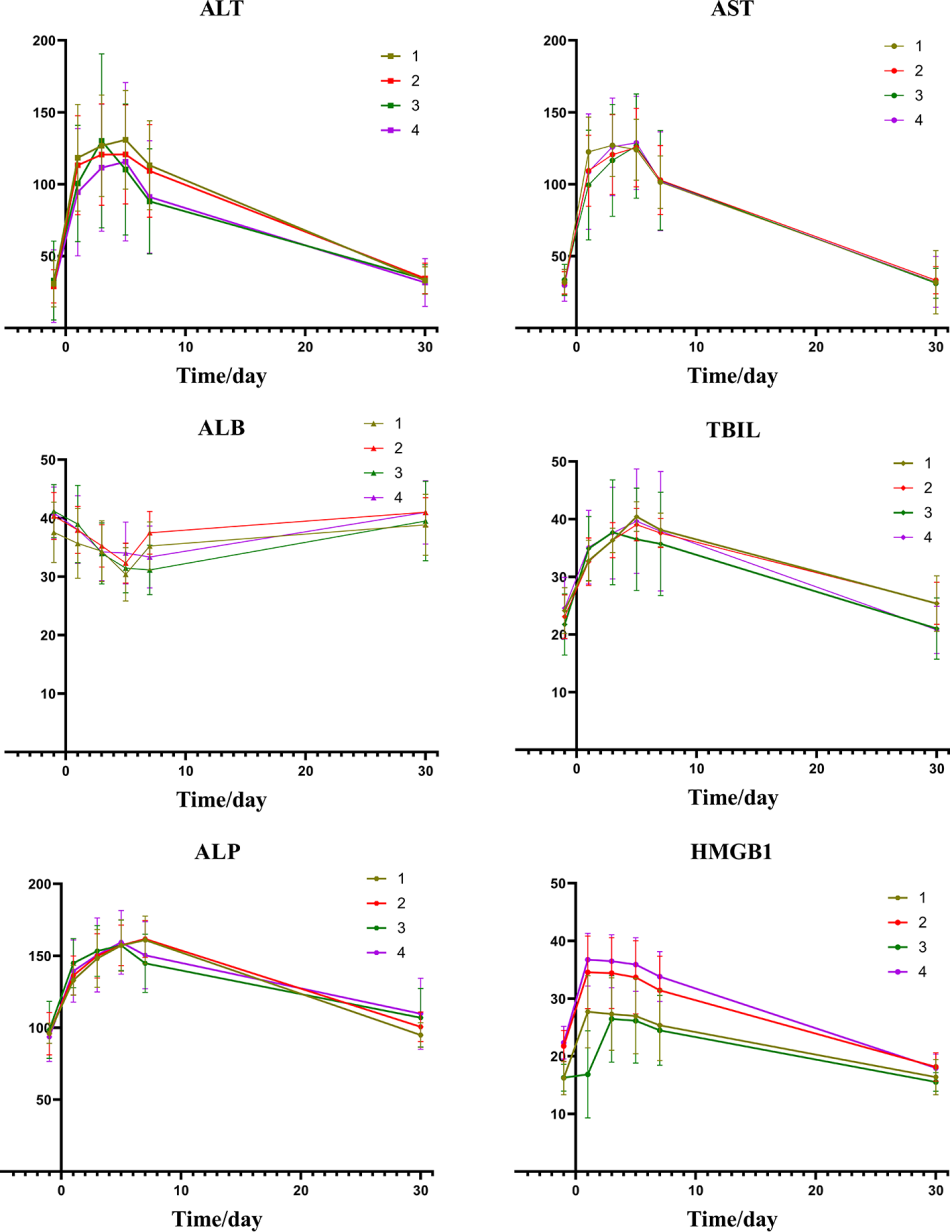
Group1: low pre-expression of HMGB1 with c-TACE; Group2: high pre-expression of HMGB1 with c-TACE; Group3: low pre-expression of HMGB1 with DEB-TACE; Group4: high pre-expression of HMGB1 with DEB-TACE.

A comparison of the changes in liver function during treatment in each group (**Figure 1**) revealed that most of the index markers failed to show sufficient statistical significance (**Table 3**), but the liver damage seems more severe in the high pre-TACE HMGB1 expression group, regardless of whether they received DEB-TACE or c-TACE.

**Table 3:** Changes of HMGB1, liver function, tumor markers and immune cells after TACE.

	DEB-TACE				c-TACE			
	High HMGB1 expression	Low HMGB1 expression	*p*-value	95%CI	High HMGB1 expression	Low HMGB1 expression	*p*-value	95%CI
Pre-ALT	29.14 ± 25.26	33.12 ± 27.48	0.58	3.97(−10.37–18.31)	29.07 ± 11.55	30.82 ± 16.12	0.66	1.75(−6.11–9.62)
1st post-ALT	94.48 ± 44.22	100.57 ± 40.44	0.61	6.09(−17.39–29.56)	113.21 ± 34.44	118.42 ± 37.04	0.61	5.21(−15.19–25.60)
3rd post-ALT	111.59 ± 44.25	130.13 ± 60.53	0.19	18.53(−9.55–46.62)	120.67 ± 35.33	126.74 ± 35.32	0.55	6.07(−14.17–26.30)
5th post-ALT	115.69 ± 55.04	110.23 ± 45.58	0.70	−5.47(−33.75–22.82)	120.77 ± 34.39	130.92 ± 34.28	0.30	10.15(−9.52–29.83)
7th post-ALT	91.17 ± 39.10	88.15 ± 36.50	0.77	−3.02(−23.93–17.88)	109.31 ± 32.20	113.28 ± 30.96	0.66	3.97(−14.17–22.11)
30th post-ALT	31.74 ± 16.61	33.94 ± 10.18	0.58	2.20(−5.73–10.13)	34.57 ± 10.60	33.07 ± 9.56	0.61	−1.49(−7.31–4.32)
Pre-AST	29.80 ± 11.08	33.49 ± 10.87	0.23	3.70(−2.34–9.73)	31.54 ± 7.73	32.08 ± 8.88	0.82	0.53(−4.20–5.26)
1st post-AST	108.78 ± 40.11	99.49 ± 38.18	0.39	−9.30(−30.89–12.30)	109.40 ± 24.73	122.55 ± 24.09	0.07	13.14(−0.86–27.15)
3rd post-AST	126.04 ± 33.92	116.64 ± 38.83	0.34	−9.40(−29.10–10.30)	120.69 ± 27.76	127.07 ± 21.70	0.38	6.37(−8.12–20.86)
5th post-AST	128.75 ± 32.42	126.58 ± 36.26	0.82	−2.17(−20.80–16.64)	125.54 ± 27.37	124.07 ± 21.24	0.84	−1.47(−15.71–12.78)
7th post-AST	102.14 ± 34.33	102.74 ± 34.61	0.95	0.60(−18.29–19.49)	103.01 ± 23.99	101.51 ± 18.19	0.81	−1.50(−13.50–10.90)
30th post-AST	32.10 ± 17.73	31.23 ± 10.43	0.82	−0.87(−8.42–6.69)	33.33 ± 9.46	31.94 ± 22.00	0.63	−1.39(−7.21–4.44)
Pre-ALB	40.89 ± 4.43	41.22 ± 4.54	0.78	0.34(−2.12–2.79)	40.37 ± 4.01	37.60 ± 5.17	0.04	−2.77(−5.37–4.57)
1st post-ALB	38.07 ± 5.78	39.00 ± 6.61	0.58	0.93 (−2.42–4.28)	37.97 ± 4.01	35.70 ± 5.97	0.11	−2.27(−5.12–−0.57)
3rd post-ALB	34.26 ± 4.95	33.98 ± 5.22	0.84	−0.28(−3.05–2.50)	35.28 ± 3.62	34.36 ± 5.19	0.46	−0.93(−3.43–1.58)
5th post-ALB	34.05 ± 5.29	31.46 ± 4.22	0.06	−2.59(−5.28–0.10)	32.33 ± 3.40	30.41 ± 4.57	0.10	−1.92(−4.18–0.35)
7th post-ALB	33.36 ± 5.31	31.17 ± 4.23	0.11	−2.19(−4.89–0.51)	37.50 ± 3.65	35.24 ± 4.11	0.04	−2.26(−4.47–−0.05)
30th post-ALB	41.00 ± 5.41	39.53 ± 6.82	0.37	−1.46(−4.75–1.83)	41.04 ± 2.49	38.87 ± 5.22	0.08	−2.17(−4.64–0.31)
Pre-TBIL	24.57 ± 5.34	21.76 ± 5.33	0.06	−2.82(−5.75–0.11)	23.10 ± 3.77	24.15 ± 3.97	0.35	1.04(−1.17–3.25)
1st post-TBIL	35.14 ± 6.39	34.91 ± 5.56	0.89	−0.23(−3.57–3.10)	32.64 ± 4.12	32.83 ± 3.54	0.86	1.10(−2.02–2.41)
3rd post-TBIL	37.61 ± 7.97	37.73 ± 9.09	0.96	0.12(−4.50–4.74)	36.37 ± 3.06	36.32 ± 2.12	0.95	−0.05(−1.59–1.49)
5th post-TBIL	39.66 ± 9.07	36.52 ± 8.89	0.21	−3.14(−8.08–1.79)	39.05 ± 2.79	40.46 ± 2.57	0.07	1.40(−.0.14–2.95)
7th post-TBIL	37.92 ± 10.35	35.72 ± 8.96	0.42	−2.20(−7.60–3.19)	37.61 ± 2.51	38.16 ± 2.91	0.48	0.55(−0.99–2.09)
30th post-TBIL	20.81 ± 4.10	21.05 ± 5.32	0.85	0.24(−2.30–2.77)	25.42 ± 3.66	25.36 ± 4.81	0.96	−0.06(−2.47–2.35)
Pre-ALP	93.63 ± 17.09	98.52 ± 19.79	0.30	4.89(−4.43–14.20)	95.84 ± 14.74	96.20 ± 6.99	0.91	0.36(−6.50–7.23)
1st post-ALP	139.34 ± 21.65	144.91 ± 17.02	0.31	5.58(−5.38–16.54)	136.35 ± 13.60	133.06 ± 10.40	0.35	−3.29(−10.34–3.76)
3rd post-ALP	150.61 ± 25.76	153.41 ± 17.67	0.63	2.79(−8.85–14.44)	149.94 ± 15.35	148.18 ± 20.17	0.73	−1.76(−11.85–8.34)
5th post-ALP	159.43 ± 22.13	157.26 ± 17.56	0.70	−2.18(−13.41–9.05)	157.31 ± 14.06	157.13 ± 17.90	0.97	−0.18(−12.25–8.74)
7th post-ALP	150.40 ± 23.28	144.74 ± 20.31	0.36	−5.66(−17.82–6.51)	161.76 ± 12.85	161.03 ± 16.70	0.86	−0.73(−9.13–7.67)
30th post-ALP	109.69 ± 24.76	106.94 ± 20.36	0.67	−2.75(−15.44–9.95)	100.65 ± 10.29	94.91 ± 8.61	0.04	−5.74(−11.23–−0.24)
Pre-CEA	547.90 ± 1,506.18	369.10 ± 646.92	0.60	−178.80(−861.57–503.97)	433.67 ± 444.94	542.61 ± 1139.32	0.65	108.95(−363.17–581.06)
30th post-CEA	416.60 ± 1,120.93	247.60 ± 409.36	0.86	20.63(−204.03–245.29)	204.43 ± 206.56	214.52 ± 318.59	0.89	10.09(−139.74–159.91)
Pre-CA19-9	547.39 ± 1,777.83	416.60 ± 1120.93	0.76	−130.78(−984.19–722.62)	565.02 ± 890.67	592.34 ± 1437.19	0.94	27.32(−638.22–692.86)
30th post- CA19-9	137.11 ± 234.16	286.40 ± 712.42	0.35	149.28(−174.99–473.56)	330.32 ± 567.47	161.34 ± 231.36	0.20	−168.99(−428.07–70.09)
Pre-CD3+	64.45 ± 7.46	66.94 ± 9.76	0.29	2.49(−2.13–7.11)	64.65 ± 13.57	65.42 ± 8.63	0.82	0.78(−5.91–7.46)
7th post-CD3+	56.42 ± 5.21	57.09 ± 5.24	0.64	0.66(−2.20–3.53)	58.43 ± 5.59	58.87 ± 5.09	0.78	0.43(−2.65–3.51)
30th post-CD3+	66.02 ± 7.10	67.68 ± 8.93	0.45	1.66(−2.66–5.97)	67.58 ± 8.78	66.40 ± 7.30	0.62	−1.17(−5.85–3.50)
Pre-CD19+	11.55 ± 3.51	11.48 ± 3.90	0.94	−0.07(−2.09–1.84)	11.44 ± 4.05	9.88 ± 2.41	0.09	−1.56(−3.42–0.29)
7th post–CD19+	10.19 ± 3.15	10.13 ± 3.41	0.94	−0.06(−1.85–1.72)	10.05 ± 3.34	8.31 ± 2.25	0.03	−1.75(−3.34–−0.15)
30th post–CD19+	11.94 ± 3.36	11.94 ± 3.83	0.99	−0.01(−1.95–1.95)	11.75 ± 3.89	10.15 ± 2.44	0.08	−1.59(−3.40–0.22)
Pre-NK cell	24.89 ± 8.79	27.03 ± 8.04	0.36	2.14(−2.53–6.81)	25.41 ± 9.34	25.38 ± 8.20	0.70	0.97(−4.11–6.04)
7th post-NK cell	20.18 ± 7.69	22.54 ± 7.92	0.27	2.37(−1.90–6.63)	19.43 ± 7.68	20.21 ± 7.14	0.71	0.79(−3.48–5.05)
30th post-NK cell	24.93 ± 8.76	27.50 ± 7.98	0.27	2.57(−2.07–7.21)	24.65 ± 9.16	25.55 ± 7.98	0.72	0.90(−4.026–5.86)
Pre-CD3+CD4+	39.67 ± 6.70	37.24 ± 5.87	0.17	−2.43(−5.94–1.07)	36.47 ± 6.77	39.07 ± 8.56	0.24	2.60(−1.76–6.96)
7th post-CD3+CD4+	43.10 ± 6.31	40.55 ± 7.22	0.17	−2.55(-6.21–1.12)	41.94 ± 6.54	44.24 ± 6.50	0.22	2.30(−1.43–6.04)
30th post-CD3+CD4+	39.95 ± 7.37	38.11 ± 7.78	0.38	−1.85(−5.98–2.29)	36.16 ± 6.78	39.04 ± 8.99	0.20	2.89(−1.59–7.37)
Pre-CD3+CD8+	28.23 ± 4.38	29.73 ± 4.22	0.21	1.50(−0.87–3.87)	26.75 ± 6.66	28.17 ± 5.38	0.42	1.41(−2.09–4.93)
7th post-CD3+CD8+	26.62 ± 4.37	27.88 ± 3.39	0.26	1.26(−0.94–3.47)	24.91 ± 6.30	26.30 ± 4.95	0.39	1.40(−1.90–4.69)
30th post-CD3+CD8+	28.59 ± 4.03	29.46 ± 4.40	0.45	0.87(−1.42–3.16)	26.54 ± 6.43	28.18 ± 5.16	0.34	1.64(−1.74–5.02)
Pre-CD4/CD8	1.43 ± 0.30	1.27 ± 0.25	0.04	−0.16(−0.36–−0.01)	1.41 ± 0.30	1.42 ± 0.37	0.89	0.013(−0.18–0.20)
7th post-CD4/CD8	1.65 ± 0.33	1.47 ± 0.32	0.04	−0.18(−0.36–−0.01)	1.76 ± 0.39	1.73 ± 0.38	0.79	−.03(−0.25–0.19)
30th post-CD4/CD8	1.41 ± 0.28	1.33 ± 0.38	0.35	−0.08(−0.26–0.11)	1.41 ± 0.30	1.41 ± 0.34	0.97	0.00(−0.18–0.19)

**Figure 2:**
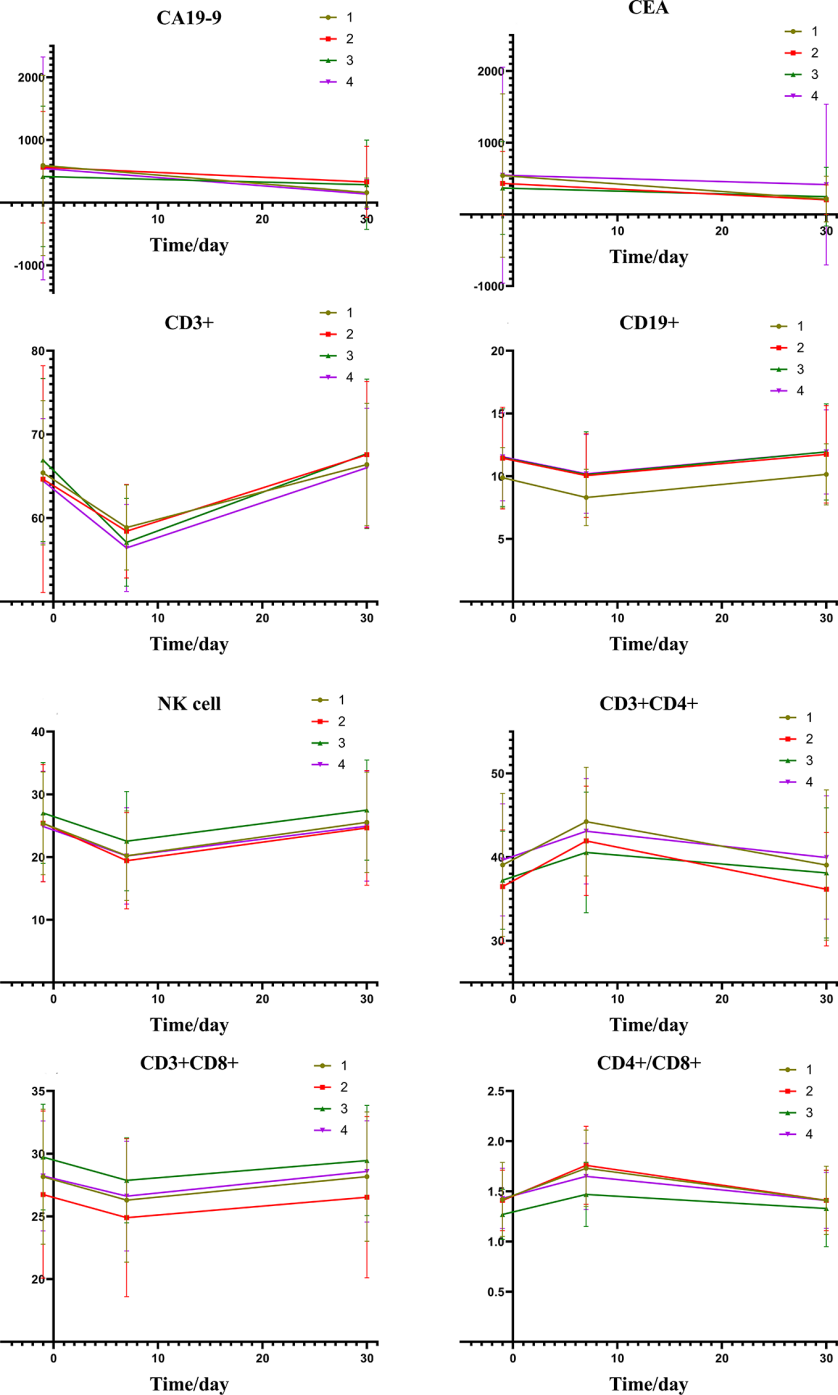
Group1: low pre-expression of HMGB1 with c-TACE; Group2: high pre-expression of HMGB1 with c-TACE; Group3: low pre-expression of HMGB1 with DEB-TACE; Group4: high pre-expression of HMGB1 with DEB-TACE.

**Figure 2** depicts the changes of tumor markers and immune function of patients during the treatment, which have more detailed comparisons in **Table 3**. There was a significant decrease of tumor marker in all patients after treatment. The patients in the four groups had transient immune disorders after TACE, but the degree of inhibition in the low expression group was slight than the others. Immune function of all could be recovered to the level of pre-treatment for one month.

The results of statistics of some common adverse reactions rate after TACE are shown in **Table 4**. Patients had similar risks of vomiting, abdominal pain, and nausea, but those who exhibited high expression of HMGB1 before TACE had a higher risk of fever.

**Table 4:** Expression of HMGB1 group, common adverse reactions treatment outcomes.

	DEB-TACE			c-TACE		
	High HMGB1 expression (34)	Low HMGB1 expression (22)	*p*-value	High HMGB1 expression (28)	Low HMGB1 expression (22)	*p*-value
fever	19	5	0.01	20	9	0.03
vomit	15	6	0.20	11	5	0.22
nausea	20	9	0.19	15	8	0.23
abdominal pain	17	7	0.19	12	6	0.26
hepatic failure	0	0	0	0	0	0
CR ^a^	2	4		1	3	
PR ^b^	16	14		14	15	
SD ^c^	14	3		10	4	
PD ^d^	2	1		3	0	
ORR ^e^	18	18	0.02	15	18	0.03
Pre-score of Qol^f^	32.76	33.10		31.72	20.91	
Post-score of Qol	47.91	48.32		48.29	47.86	

^a^CR, complete response.

^b^PR, partial response.

^c^SD, stable disease.

^d^PD, progressive disease.

^e^ORR, Objective response rate; ORR = CR + PR.

^f^Qol, quality of life.

The analysis of the treatment outcomes of the four groups of patients is shown in **Table 4**. Combined with the significant decrease of tumor markers, it can be found that most patients have a good treatment effect even if they receive different TACE. The table also shows that the quality of life of all patients was significantly improved after TACE.

**Figure 3:**
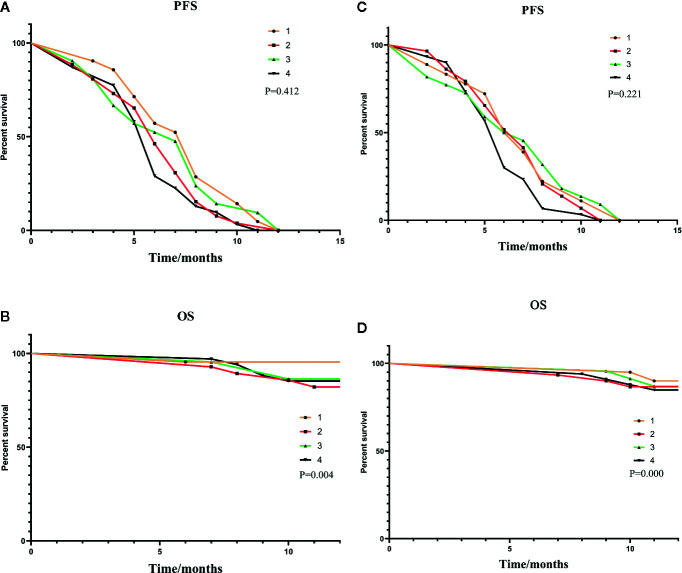
**(A, B)** were subgroup analyses of HMGB1 expression before TACE, and **(C, D)** were subgroup analyses based on HMGB1 changes after TACE. In **(A, B)**: Group1: low pre-expression of HMGB1 with c-TACE; Group2: high pre-expression of HMGB1 with c-TACE; Group3: low pre-expression of HMGB1 with DEB-TACE; Group4: high pre-expression of HMGB1 with DEB-TACE. In **(C, D)**:Group1: low post-expression of HMGB1 with c-TACE; Group2: high post-expressions of HMGB1 with c-TACE; Group3: low post-expressions of HMGB1 with DEB-TACE; Group4: high post-expression of HMGB1 with DEB-TACE.

As shown in **Figure 3A**, a correlation was found between the pre-expression of HMGB1 and PFS. The differences between the level of HMGB1 and 1-year survival are highlighted in **Figure 3B**.

### The Change of Post-TACE Level of HMGB1 and Prognosis

The basic information about the four groups of patients is shown in **Table 3**.

**Figure 4:**
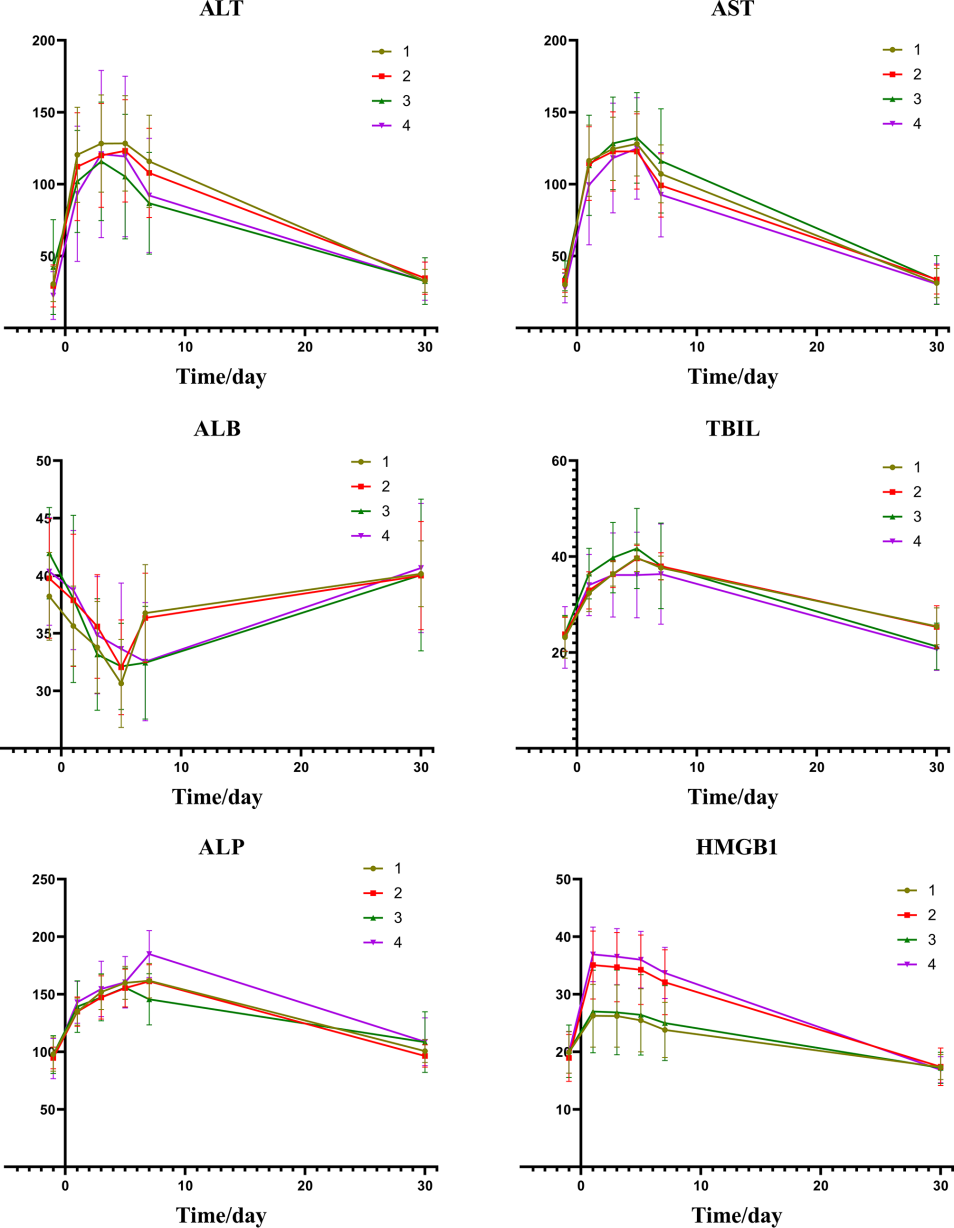
Group1: low post-expression of HMGB1 with c-TACE; Group2: high post-expression of HMGB1 with c-TACE; Group3: low post-expression of HMGB1 with DEB-TACE; Group4: high post-expression of HMGB1 with DEB-TACE.

From the information of **Figures 4** and **Table 5**, we can find that there is a certain relation between the changes of liver function and the changes of HMGB1. The rise of HMGB1 is accompanied by subsequent liver function damage, which means that the rise of HMGB1 probably indicates the severity of the liver injury. It can be seen from the change trend chart that as the change curve of HMGB1 showed a significant rise, the markers of liver damage also showed a significant upward trend in the following days.

**Table 5:** Post-expression of HMGB1 group and changes of liver function, tumor marker and immune after TACE.

	DEB-TACE				c-TACE			
	High-change(n = 33)	Low-change (n = 23)	*p*-value	95%CI	High-change (n = 30)	Low-change (n = 20)	*p*-value	95%CI
Pre-ALT	22.57 ± 16.67	42.38 ± 32.99	0.01	19.82(4.68–34.95)	29.28 ± 14.62	30.68 ± 12.30	0.73	1.40 (−6.58–9.38)
1st post-ALT	93.37 ± 47.00	101.89 ± 35.49	0.44	8.52(−13.60–30.64)	112.22 ± 37.52	120.41 ± 33.07	0.43	8.19(−12.40–28.77)
3rd post-ALT	120.93 ± 58.19	115.92 ± 41.25	0.72	−5.01(−33.30–23.29)	120.06 ± 36.13	128.26 ± 33.78	0.42	8.21(−12.24–28.65)
5th post-ALT	119.32 ± 55.90	105.26 ± 43.34	0.32	−14.05(−41.91–13.81)	123.15 ± 35.59	128.37 ± 33.11	0.60	5.22 (−14.88–25.32)
7th post-ALT	92.20 ± 39.77	86.80 ± 35.37	0.60	−5.40(−26.12–15.32)	107.82 ± 31.02	115.91 ± 32.15	0.38	8.09(10.18–26.35)
30th post-ALT	32.54 ± 13.20	32.69 ± 16.20	0.97	0.15(−7.75–8.04)	34.65 ± 11.30	32.80 ± 8.05	0.53	−1.84 (−7.73–4.04)
Pre-AST	27.75 ± 10.27	36.26 ± 10.36	0.00	8.51(2.90–14.12)	32.84 ± 8.07	30.19 ± 8.28	0.27	−2.64 (−7.38–2.09)
1st post-AST	99.52 ± 41.65	113.17 ± 34.92	0.20	13.65(−7.62–34.91)	114.42 ± 25.65	116.34 ± 24.81	0.79	1.92 (−12.78–16.62)
3rd post-AST	118.17 ± 38.13	128.34 ± 32.24	0.30	10.17(−9.35–29.69)	122.80 ± 27.52	124.55 ± 22.01	0.81	1.75(−13.04–16.54)
5th post-AST	124.91 ± 35.29	132.18 ± 31.47	0.43	7.27(−11.13–25.67)	122.78 ± 26.18	128.07 ± 22.38	0.46	5.29 (−9.07–19.65)
7th post-AST	92.71 ± 29.33	116.25 ± 36.31	0.01	23.54(5.92–41.16)	99.11 ± 22.03	107.20 ± 20.07	0.19	8.09(−4.26–20.44)
30th post-AST	30.60 ± 14.00	33.42 ± 16.91	0.50	2.82(−5.48–11.13)	33.67 ± 10.08	31.29 ± 10.18	0.42	−2.38(−8.25–3.50)
Pre-ALB	40.37 ± 4.68	41.96 ± 3.97	0.19	1.59(−0.81–3.99)	39.78 ± 5.20	38.22 ± 3.82	0.26	−1.56 (−4.29–1.16)
1st post-ALB	38.76 ± 5.18	37.99 ± 7.27	0.64	−0.77(−4.10–2.56)	37.87 ± 5.75	35.63 ± 3.47	0.13	−2.24(−5.12–0.65)
3rd post-ALB	34.84 ± 5.10	33.16 ± 4.84	0.22	−1.68(-4.40–1.04)	35.60 ± 4.51	33.78 ± 3.98	0.15	−1.83(−4.32–0.67)
5th post-ALB	33.65 ± 5.72	32.13 ± 3.75	0.27	−1.52(−4.25–1.21)	32.05 ± 4.12	30.64 ± 3.83	0.23	−1.41(−3.74–0.92)
7th post-ALB	32.54 ± 5.14	32.45 ± 4.90	0.95	−0.09(−2.84–2.65)	36.34 ± 3.89	36.76 ± 4.21	0.72	0.43(−1.91–2.76)
30th post-ALB	40.67 ± 5.62	40.07 ± 6.59	0.71	−0.60(−3.89–2.68)	40.02 ± 4.71	40.17 ± 2.86	0.90	0.15(−2.22–2.51)
Pre-TBIL	23.14 ± 6.42	23.94 ± 3.80	0.56	0.80(−1.95–3.54)	23.82 ± 3.54	23.18 ± 4.35	0.57	−0.64 (−2.90–1.61)
1st post-TBIL	34.07 ± 6.38	36.46 ± 5.30	0.15	2.39(−0.85–5.64)	32.95 ± 3.89	32.38 ± 3.83	0.61	−0.57 (−2.81–1.67)
3rd post-TBIL	36.17 ± 8.78	39.79 ± 7.34	0.11	3.62(−0.86–8.09)	36.31 ± 2.78	36.40 ± 2.56	0.91	0.09 (−1.47–1.65)
5th post-TBIL	36.15 ± 8.93	41.70 ± 8.36	0.02	5.54(0.80–10.28)	39.61 ± 2.73	39.76 ± 2.87	0.85	0.16(−1.46–1.77)
7th post-TBIL	36.35 ± 10.44	38.07 ± 8.93	0.52	1.71(−3.65–7.08)	37.98 ± 2.85	37.66 ± 2.46	0.68	−0.32 (−1.89–1.25)
30th post-TBIL	20.65 ± 4.41	21.27 ± 4.88	0.62	0.62(−1.89–3.13)	25.34 ± 4.41	25.47 ± 3.86	0.91	0.13 (−2.31–2.57)
Pre-ALP	94.15 ± 17.51	97.56 ± 16.39	0.47	3.40(−5.89–12.69)	94.67 ± 9.37	97.99 ± 14.90	0.34	3.32 (−3.57–10.21)
1st post-ALP	143.07 ± 18.30	139.31 ± 22.43	0.49	−3.76(−14.69–7.18)	134.86 ± 12.76	134.98 ± 11.87	0.97	0.12(−7.09–7.32)
3rd post-ALP	154.71 ± 24.12	147.41 ± 20.49	0.24	−7.29(−19.66–5.07)	147.28 ± 18.80	151.99 ± 15.26	0.36	4.72(−5.43–14.87)
5th post-ALP	160.50 ± 22.40	155.82 ± 16.97	0.40	−4.68(−15.77–6.42)	155.41 ± 16.49	159.97 ± 14.39	0.32	4.57(−4.55–13.67)
7th post-ALP	184.98 ± 204.77	145.59 ± 22.23	0.36	−39.40(−125.59–46.80)	161.20 ± 14.43	161.80 ± 15.00	0.89	0.61(−7.90–9.11)
30th post-ALP	108.72 ± 20.79	108.44 ± 26.29	0.96	−0.28(−12.91–12.34)	96.42 ± 9.67	100.69 ± 9.97	0.14	4.27 (−1.41–9.96)
Pre-CEA	605.42 ± 1539.29	294.36 ± 561.91	0.36	−311.28(−985.27–363.15)	494.96 ± 1014.14	461.57 ± 391.46	0.89	−33.39(−512.73–445.95)
30th post-CEA	263.92 ± 472.67	193.69 ± 289.73	0.53	−70.23(−292.51–152.04)	205.88 ± 304.64	213.35 ± 176.62	0.92	7.48(−144.34–159.30)
Pre-CA19-9	569.49 ± 1806.03	390.58 ± 1089.37	0.67	−178.90(−1025.43–667.63)	666.36 ± 1466.61	440.06 ± 307.87	0.50	−228.30(−899.45–442.85)
30th post- CA19-9	160.49 ± 253.45	246.37 ± 694.45	0.52	85.89(−177.85–349.62)	286.07 ± 569.82	210.82 ± 190.05	0.57	−75.24(−341.52–191.04)
Pre-CD3+	65.77 ± 9.25	64.94 ± 7.30	0.72	−0.83(−5.47–3.80)	63.71 ± 12.82	66.91 ± 9.33	0.34	3.20(−3.52–9.91)
7th post-CD3+	56.30 ± 4.72	57.24 ± 5.85	0.51	0.94(−1.90–3.77)	58.46 ± 5.41	59.87 ± 5.32	0.79	0.41(−2.71–3.53)
30th post-CD3+	66.64 ± 8.39	66.72 ± 7.14	0.97	0.08(−4.23–4.38)	66.65 ± 8.34	67.67 ± 7.91	0.67	1.02(−3.72–5.76)
Pre-CD19+	11.72 ± 3.71	11.24 ± 3.60	0.64	−0.47(−2.47–1.52)	10.60 ± 3.30	10.98 ± 3.81	0.71	0.38(−1.66–2.42)
7th post–CD19+	10.32 ± 3.26	9.94 ± 3.24	0.67	−0.38(−2.15–1.39)	9.10 ± 2.84	9.56 ± 3.31	0.60	0.46(−1.30–2.23)
30th post–CD19+	12.13 ± 3.62	11.66 ± 3.44	0.62	−0.47(−2.41–1.46)	10.91 ± 3.34	11.25 ± 3.55	0.73	0.35(−1.64–2.34)
Pre-NK cell	25.72 ± 8.83	25.75 ± 8.20	0.99	0.03(−4.64–4.70)	25.41 ± 9.44	23.98 ± 7.85	0.58	−1.43(−6.56–3.70)
7th post-NK cell	20.92 ± 7.88	21.37 ± 7.85	0.84	0.45(−3.84–4.73)	20.37 ± 8.14	18.87 ± 6.14	0.46	−1.51(−5.57–2.57)
30th post-NK cell	25.95 ± 8.75	25.93 ± 8.28	0.99	−0.03(−4.69–4.63)	25.58 ± 8.94	24.26 ± 8.18	0.60	−1.31(−6.33–3.71)
Pre-CD3+CD4+	38.23 ± 6.50	39.41 ± 6.43	0.51	1.17(−2.36–4.70)	37.21 ± 6.97	38.23 ± 8.71	0.65	1.02(−3.45–5.49)
7th post-CD3+CD4+	42.00 ± 6.69	42.24 ± 6.95	0.90	0.24(−3.46–3.95)	42.97 ± 6.04	42.93 ± 7.44	0.98	−0.04(−3.89–3.81)
30th post-CD3+CD4+	38.47 ± 7.26	40.31 ± 7.91	0.37	1.85(−2.26–5.95)	37.22 ± 7.42	37.74 ± 8.71	0.82	0.52(−4.09–5.14)
Pre-CD3+CD8+	29.19 ± 3.94	28.28 ± 4.89	0.44	−0.91(−3.28–1.46)	26.94 ± 6.05	28.02 ± 6.30	0.55	1.08(−2.49–4.65)
7th post-CD3+CD8+	27.37 ± 3.54	26.76 ± 4.70	0.58	−0.61(−2.82–1.60)	25.33 ± 6.05	25.81 ± 6.03	0.77	0.48(−2.87–3.84)
30th post-CD3+CD8+	29.12 ± 3.77	28.67 ± 4.74	0.70	−0.45(−2.73–1.84)	26.87 ± 5.82	27.85 ± 6.13	0.57	0.98(−2.47–4.43)
Pre-CD4/CD8	1.32 ± 0.21	1.44 ± 0.37	0.17	0.12(−0.05–0.29)	1.44 ± 0.40	1.28 ± 0.20	0.46	−0.06(−0.23–0.11)
7th post-CD4/CD8	1.55 ± 0.27	1.63 ± 0.41	0.40	0.08(−0.11–0.26)	1.77 ± 0.42	1.71 ± 0.33	0.62	−0.06(−0.28–0.17)
30th post-CD4/CD8	1.33 ± 0.24	1.45 ± 0.40	0.21	0.12(−0.07–0.31)	1.43 ± 0.36	1.37 ± 0.23	0.48	−0.07(−0.25–0.12)

**Figure 5:**
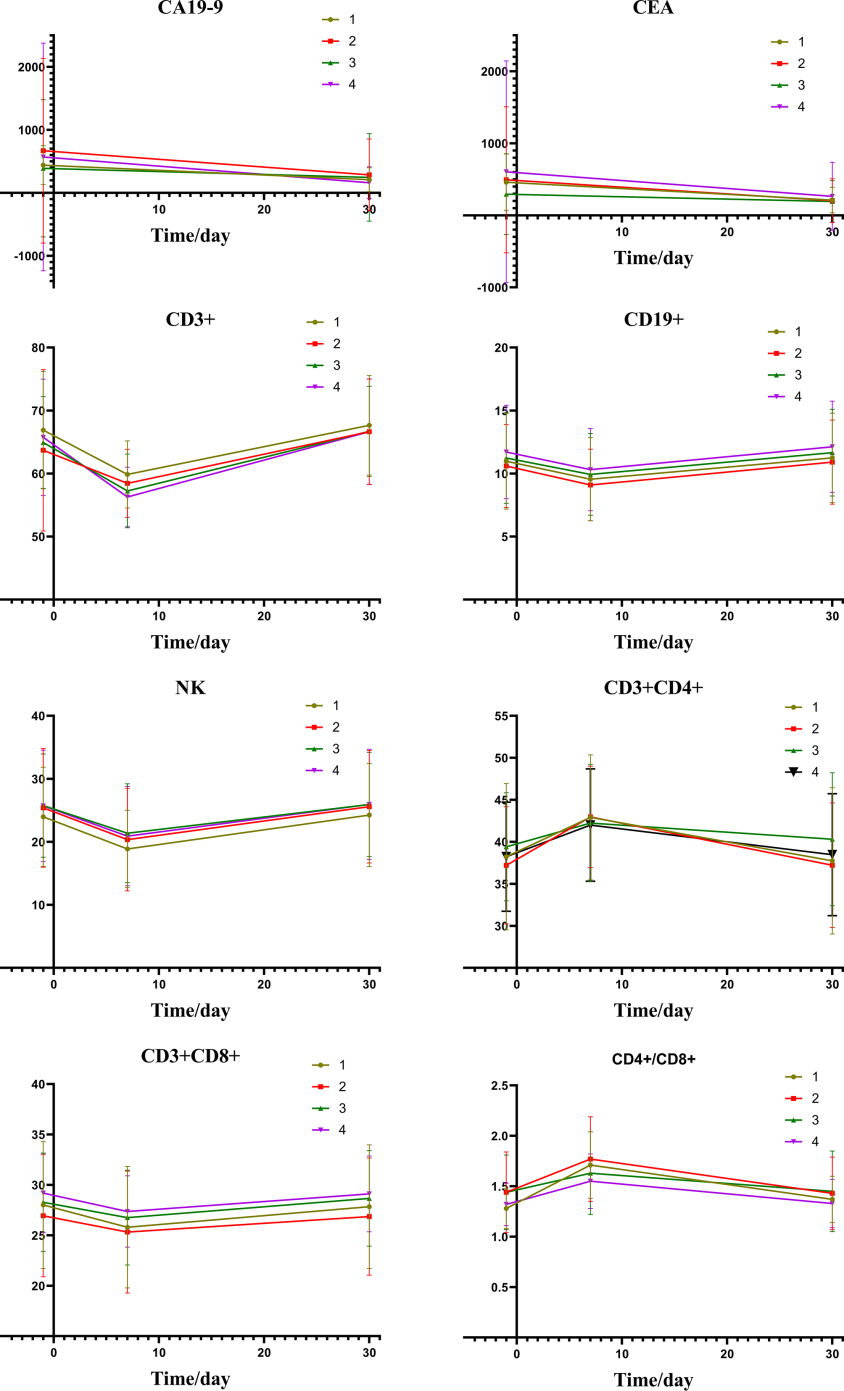
Group1: low post-expression of HMGB1 with c-TACE; Group2: high post-expression of HMGB1 with c-TACE; Group3: low post-expression of HMGB1 with DEB-TACE; Group4: high post-expression of HMGB1 with DEB-TACE.

**Table 5** shows the changes of tumor markers and immune function after TACE. The trend chart (**Figure 5**) shows that the degree of immunosuppression after DEB-TACE was slightly severer than that after c-TACE; however, the difference between them revealed no statistically significant differences.

The adverse reactions after the TACE of the four groups were analyzed, and the results are shown in **Table 6**. Fever is the most closely related adverse reaction to the change of HMGB1.

**Table 6:** Post-expression of HMGB1group, common adverse reactions and treatment outcomes.

	DEB-TACE			c-TACE		
	High-change (33)	Low-change (23)	p-value	High-change (30)	Low-change (20)	*p*-value
fever	18	6	0.04	21	8	0.03
vomit	11	10	0.45	10	6	0.81
nausea	18	11	0.63	11	12	0.11
abdominal pain	14	10	0.94	10	8	0.64
hepatic failure	0	0	0	0	0	0
CR ^a^	3	3		2	2	
PR ^b^	13	17		14	15	
SD ^c^	15	2		12	2	
PD ^d^	2	1		2	1	
ORR ^e^	16	20	<0.01	16	17	0.01
Pre-score of Qol ^f^	32.64	33.26		31.27	31.50	
Post-score of Qol	47.58	48.78		48.37	47.70	

^a^CR, complete response.

^b^PR, partial response.

^c^SD, stable disease.

^d^PD, progressive disease.

^e^ORR, Objective response rate; ORR = CR + PR.

^f^Qol, quality of life.

The treatment results of the four groups are shown in **Table 6**. The patients with a small increase in HMGB1 after TACE have a relatively good treatment effect. The PFS and one-year survival are shown in **Figures 3C, D**.

As can be seen from the data in **Table 6**, the quality of life of all patients benefits from treatment.

## Discussion

In this study, the level of HMGB1 was found significantly elevated in the blood after TACE. It expands the knowledge on the association between HMGB1 and treatment outcome in LMCRC by showing its magnitude rather than just showing that there is a statistically significant relationship. HMGB1 was more enriched in the serum of patients with severe liver impairment compared to the preoperative low-expression group. We identified a highly significant relation among HMGB1expression, liver damage, and PFS.

In addition, the analysis of the changes of HMGB1 after TACE can improve the sensitivity of it in the diagnosis of liver function damage. HMGB1 may be a possible prognostic factor for adverse reactions in patients with LMCRC.

Due to their stability and specificity in most bodily fluids, HMGB1 provides a high potential to serve as a liquid biopsy tool for some cancers and sterile inflammation (18). Some researchers performed proteomic analyses to the clinical significance of HMGB1 in serum-purified exosomes from malignant mesothelioma cancer patients and identified it as potential biomarkers in diagnosis and prognosis (19, 20). Dr. Venereau believes that high levels of serum hyper-acetylated HMGB1 are sensitive disease biomarkers (21). He also found that injection of HMGB1 accelerates tissue repair by acting on muscle stem cells, hepatocytes, and infiltrating cells (22). Dr. Liu concludes that HMGB1 protein is a valuable marker for the progression of CRC patients. High HMGB1 expression is associated with poor overall survival in patients with CRC (23).

In the case of LMCRC, we have further confirmed strong correlations between elevated expression of HMGB1 and liver damage or PFS and discovered strong correlations between elevated the HMGB1 and the effect of palliative treatment including the c-TACE and DEB-TACE.

This study focused on the expression and changes of HMGB1 in the serum of colorectal cancer patients with liver metastasis before and after TACE, and we explored the possibility of predicting liver injury, adverse reaction, and PFS after TACE by monitoring the changes of HMGB1. In this study, we detected and compared the expression of HMGB1 before TACE and on the first, third, fifth, and seventh days after TACE. The results of data analysis and change trend chart showed that the expression of HMGB1 changed significantly and reached the peak on the 1st day after TACE, and the expression was stable on the 3rd, 5th, and 7th days. The expression of HMGB1 returned to pre-TACE until the 30th day. For those with low expression of HMGB1 before TACE, the liver injury was slighter, while for those with the severe rise of HMGB1 after treatment, the liver injury was more serious. The expression level of HMGB1 was positively correlated with the degree of liver injury. This result suggests that the level of HMGB1 on the 1st day after treatment compared with pre-TACE expression may predict liver injury.

Embolization and reperfusion after TACE are a standard process of liver ischemia–reperfusion injury in patients (13). After complete embolization of the target vessel by the doctor, the tumor focus of the liver and the surrounding normal liver tissue will form a temporary ischemic area (13). The interruption of the blood supply has left the area in a state of ischemia, hypoxia, and nutrient deprivation, with a large number of tumor cells and hepatocyte death (24). However, the thrombolytic effect of TACE gradually declined with the subsequent constant flushing of blood from the ischemic area. At the same time, collateral circulation was established in the embolized area, which made the embolized area get blood perfusion again. In the whole process, the embolization of drugs and blood vessels not only killed tumor cell, but also produced liver damage. Treatment stress and the changes of tumor microenvironment can aggravate the acute liver failure (25).

HMGB1 plays an important role in the process of ischemia–reperfusion after TACE, and its acetylation and release are mainly regulated by four main modes (18). Firstly, large amounts of hypoxanthine accumulated converted to xanthine during anaerobic respiration after the interruption of blood supply, and the resulting ROS prompt cells to release acetylated HMGB1 (26, 27). Secondly, Kupffer cells can be activated to release IL-1β, IL-6, and TNF-α, which can promote the acetylation of HMGB1 (28, 29). Thirdly, during the late stage of ischemia/reperfusion (the phase of injury caused by blood reperfusion), activated neutrophils and macrophages begin to converge and accumulate towards the ischemic area and stimulate HMGB1 release through the release of inflammatory factors. Lastly, ischemia–reperfusion can lead to Ca^+^ overload, which caused abnormal mitochondrial membrane permeability transporter pore and abnormal electron transport in the respiratory chain to produce ROS, then stimulates the local massive release of HMGB1 (30, 31). Under the combined action of various factors, a large amount of acetylated HMGB1 is released into the extracellular space, and its expression in the ischemic area and body blood rises rapidly. After TACE, the blood vessels of the tumor and surrounding tissue are embolized, which causes ischemia. However, the embolic material in the blood vessel is rinsed off by the blood, and the rapid formation of collateral vessels makes the ischemic area quickly regain blood supply. The large amount of HMGB1 produced in this process not only increased in the ischemic area but also reached the whole liver and the whole body by means of blood circulation, which leads to local and systemic inflammation of the liver (32). During ischemia–reperfusion in the liver, HMGB1 plays an important mediating role: within 1– h after TACE, cells in the ischemic area begin to necrotize and rupture, and release HMGB1. Induced by extracellular HMGB1 and other inflammatory factors, it leads to the release of more HMGB1 from the cells in the non-embolized area and mediates severe inflammatory response in the ischemic area (31, 33). Meanwhile, HMGB1 acetylated during the reperfusion phase can stimulate aggregated macrophages and monocytes to actively acetylate and release more HMGB1 (34).

According to the statistics and analysis of common adverse reactions after TACE, patients with low pre-TACE expression of HMGB1 had a lower incidence of fever than those with higher expression. In addition, patients with a lower postoperative increase in HMGB1 expression had a lower risk of developing a fever. The results suggest that there is a correlation between the expression of HMGB1 and fever. On the one hand, post-TACE fever is due to the absorption of necrotic material at the site of embolization, which is the classic “absorption heat”. On the other hand, HMGB1 can lead to the release of pro-inflammatory factors and excitatory amino acids in the microenvironment, which also promotes the development of fever in the body (35). Therefore, the high expression of HMGB1 in the serum before and after treatment may predict a higher risk of fever in patients.

DEB-TACE was modified from c-TACE. The classical c-TACE is to infuse chemotherapeutic drugs into the blood vessels of tumors, and then embolize the blood vessels with insoluble materials. The microspheres used in DEB-TACE can both adsorb drugs and serve as materials for embolization of blood vessels. Although there are some differences in the surgical procedures, the principles of the two treatments for tumors are consistent. For ischemia and hypoxia caused by c-TACE and DEB-TACE, the reperfusion injury of vascular recanalization after two TACE are consistent, and the change of HMGB1 expression level after treatment has the same trend.

During the follow-up of this subject, we found that tumor markers decreased significantly at one month after TACE. The expression changes of tumor markers in the two groups were similar, and the difference was not statistically significant, which showed that both treatment methods had good effects on liver metastasis of colorectal cancer.

Analysis of the quality of life data showed that the QoL scores increased substantially after TACE. The results show that TACE can significantly improve the quality of life.

The results of the present study showed that patients with dramatically increased HMGB1 level after TACE had a relatively poor outcome. Patients whose HMGB1 expression increased more than 50% after TACE had shorter PFS than those whose expression smaller. From the principle of TACE, the most ideal result is that the blood supply of tumors is permanently and completely blocked, combined with the killing effect of anti-tumor drugs, achieved the therapeutic purposes. However, some tumor cells survive and continue to grow after TACE due to the rapid emergence of collateral circulation and the existence of some unembolized micro-vessels. According to related studies, HMGB1 can promote the formation and development of new blood vessels (36). HMGB1 is also associated with cancer progression and immune escape, which is able to induce angiogenesis, metastasis (37). At present, HMGB1 is known to accelerate angiogenesis by 1) acting on vascular endothelium to promoting the formation of new blood vessels and neovascular network by promoting the synthesis of endothelial growth factor. 2) Acetylated HMGB1, which has been released, activates macrophages to upregulate nuclear factor kappa B, thereby promoting the synthesis and secretion of vascular endothelial growth factor and indirectly promoting the formation of new blood vessels (38, 39). 3) HMGB1 can upregulate the expression of fibroblast growth factor (FGF) (40), stimulate the secretion of platelet-derived growth factor (PDGF) (41, 42), and greatly enhance the proliferation and migration ability of endothelial cells.

Receptor for advanced glycation end products (RAGE) plays a role in tumor metastasis after binding to HMGB1 (43, 44). The C-terminus of HMGB1 can specifically bind to RAGE binding, triggering cytoplasmic signaling required for cell movement regulation and opening the molecular switches that control cytoskeletal organization (45). HMGB1/RAGE cannot only regulate the cytoskeleton to achieve cell movement, but also attract and aggregate other cells, and enhance the ability of cell aggregation and adhesion, which plays an important role in the formation of new collateral circulation after TACE. The combination of HMGB1/RAGE makes peripheral cells and smooth muscle cells aggregate to the high expression site and promotes the formation of the vascular structure. In addition, HMGB1/RAGE can regulate the expression of the BCL-2 gene (B-cell lymphoma/leukemia-2 gene) (46, 47), which is a cancer gene with the effect of inhibiting apoptosis. The anti-apoptotic effect of HMGB1/RAGE is directly related to the expression of BCL-2 (48). The multiple effects of HMGB1 enable the tumor to rapidly establish collateral circulation after the original blood supply is interrupted by embolization, which leads to the tumor to regain some vessels after TACE. When many tumor cells are necrotic, the remaining tumor cells with blood supply at the edge grow and proliferate rapidly, which leads to the progress and recurrence of local lesions.

The small size of patients’ sample in this study may have some impact on the accuracy of the results. A larger number of patients can undoubtedly increase the accuracy of the results. The results of this study need to be verified by the analysis of large samples in the future, and we hope to promote a multicenter study with a larger sample size in the next time, which can verify the predictive role of HMGB1.

Most of the patients who had better outcomes in this study are still alive. Three-year and five-year survival rates were not analyzed due to time constraints. In the future, we will continue to follow up on the enrolled patients in order to obtain complete survival data and compare their long-term survival rates.

In conclusion, the results of this study suggest that HMGB1 may serve as a marker for predicting liver injury and long-term efficacy after TACE in patients with LMCRC. The change of HMGB1 before and after TACE is significantly associated with PFS. With the help of monitoring the change of HMGB1 expression, patients’ PFS can be effectively predicted.

## Data Availability Statement

The raw data supporting the conclusions of this article will be made available by the authors, without undue reservation.

## Ethics Statement

The studies involving human participants were reviewed and approved by Shandong Cancer Hospital Ethics Committee. The patients/participants provided their written informed consent to participate in this study.

## Author Contributions

J-jH and Y-dS made the study concept and design. Y-qC, C-xW, and J-bZ acquired the data. HZ, J-zL and H-rX made the interpretation of data. HZ and Y-dS made the analysis and drafted the manuscript. All authors contributed to the article and approved the submitted version. J-jH is the guarantor.

## Funding

This work was supported by the Natural Science Foundation of Shandong Province (No. ZR2017MH095) and The Key R & D Project of Shandong Province (No. 2019GSF108166), and Cancer Interventional Therapy Research Foundation Project of China Health Promotion Foundation (HRIRF-2018-C004).

## Conflict of Interest

The authors declare that the research was conducted in the absence of any commercial or financial relationships that could be construed as a potential conflict of interest.
